# Protein Disulfide Isomerase Modulates the Activation of Thyroid Hormone Receptors

**DOI:** 10.3389/fendo.2018.00784

**Published:** 2019-01-08

**Authors:** Jessica L. O. Campos, Tabata R. Doratioto, Natalia B. Videira, Helder V. Ribeiro Filho, Fernanda A. H. Batista, Juliana Fattori, Nathalia de C. Indolfo, Marcel Nakahira, Marcio C. Bajgelman, Aleksandra Cvoro, Francisco R. M. Laurindo, Paul Webb, Ana Carolina M. Figueira

**Affiliations:** ^1^Brazilian Biosciences National Laboratory (LNBio), Brazilian Center for Research Energy and Materials (CNPEM), São Paulo, Brazil; ^2^Graduation Program of Biosciences and Bioactive Products Technology, Institute of Biology, State University of Campinas (Unicamp), São Paulo, Brazil; ^3^Institute of Chemistry (IQ), State University of Campinas (Unicamp), São Paulo, Brazil; ^4^Genomic Medicine, The Methodist Hospital Research Institute, Houston, TX, United States; ^5^Vascular Biology Laboratory, Heart Institute (InCor), School of Medicine, University of São Paulo, São Paulo, Brazil; ^6^California Institute for Regenerative Medicine, Oakland, CA, United States

**Keywords:** thyroid hormone receptor, protein disulfide isomerase, protein complexes, redox regulation, nuclear receptor signaling pathways

## Abstract

Thyroid hormone receptors (TRs) are responsible for mediating thyroid hormone (T3 and T4) actions at a cellular level. They belong to the nuclear receptor (NR) superfamily and execute their main functions inside the cell nuclei as hormone-regulated transcription factors. These receptors also exhibit so-called “non-classic” actions, for which other cellular proteins, apart from coregulators inside nuclei, regulate their activity. Aiming to find alternative pathways of TR modulation, we searched for interacting proteins and found that PDIA1 interacts with TRβ in a yeast two-hybrid screening assay. The functional implications of PDIA1—TR interactions are still unclear; however, our co-immunoprecipitation (co-IP) and fluorescence assay results showed that PDI was able to bind both TR isoforms *in vitro*. Moreover, T3 appears to have no important role in these interactions in cellular assays, where PDIA1 was able to regulate transcription of TRα and TRβ-mediated genes in different ways depending on the promoter region and on the TR isoform involved. Although PDIA1 appears to act as a coregulator, it binds to a TR surface that does not interfere with coactivator binding. However, the TR:PDIA1 complex affinity and activation are different depending on the TR isoform. Such differences may reflect the structural organization of the PDIA1:TR complex, as shown by models depicting an interaction interface with exposed cysteines from both proteins, suggesting that PDIA1 might modulate TR by its thiol reductase/isomerase activity.

## Introduction

Thyroid hormone receptors (TRs) are responsible for mediating thyroid hormone T3 and T4 (triiodothyronine and thyroxine, respectively) actions in cells (1, 2). Thyroid hormones (THs) are essential for normal development, neural differentiation, growth and metabolic regulation in mammals (3). Once secreted by the thyroid gland (mainly as T4), THs are carried in the plasma where they bind to proteins such as serum albumin, thyroxine-binding globulin and transthyretin, until they reach target tissues or cells, in which they are transported by specific carriers, resulting in the conversion of T4 into T3 (4, 5). In adults, the deficiency or excess of THs are usually associated with diseases, including hypo- and hyperthyroidism (6, 7).

TRs are encoded by two genes, THRA and THRB, located on chromosomes 17 and 3, respectively (8). Due to their alternative splicing, there are 4 major TR isoforms: TRα1 (410 aa.), TRα2 (492 aa.), TRβ1 (461 aa.), and TRβ2 (514 aa.) (4). The TRβ subtypes only differ in the N-terminal region, despite having the same actions (9–11). These isoforms diverge in expression patterns among tissues; TRα1 and TRα2 are predominantly expressed in the brain, heart, and skeletal muscle, while TRβ1 is considered ubiquitous, and TRβ2 is expressed in the brain, inner ear and retina (3, 12).

These receptors belong to the nuclear receptor (NR) superfamily, acting inside the cell nuclei as hormone-regulated transcription factors (2). TRs have three distinct domains: an N-terminal transactivation domain (NT), a central DNA binding domain (DBD), and a C-terminal ligand binding domain (LBD) (13, 14). This last domain displays a hydrophobic pocket that recognizes and binds to THs (15). Through the DBD, TRs bind to short DNA sequences, which are the thyroid hormone responsive elements (TREs) that are located in regulatory regions of target genes. Preferably, they act as heterodimers together with retinoid X receptors (RXR) (8) and in general, when bound to TREs, they adopt a conformational configuration that allows corepressor complex recruitment, thereby repressing target gene transcription. After ligand binding, TRs activate gene transcription through conformational changes that allow dissociation of corepressors and recruitment of coactivators (15, 16). Hence, TRs, together with all transcription machinery, are able to up- and downregulate target genes depending on the cell type and on the presence/absence of their cognate hormones (1). TRs also provide so-called “non-classic” actions in which other cellular proteins, apart from coregulators, can also regulate their activity. These TR-interacting proteins are functionally diverse, ranging from tumor suppressors and promoters, to cytoskeletal architecture modulators, transcription regulators and many others (12), and depending on each partner bound to TR, the cells may execute different actions.

In addition, several studies have shown the presence of TRs in the cytoplasm not just transiently but also when interacting with different cytoplasmic proteins (17–20). Moreover, TH-related signaling cascades without the presence of TRs were also reported. For example, cytosolic 3,5,3′-triiodo-L-thyronine (T3)-binding proteins (CTBPs) can execute these “non-genomic actions” (12), such as the dimeric 76 kDa rat liver cytosol protein (20), the 38 kDa human kidney cytosol protein (21), the NADPH-activated liver cytosol protein (22), and the cytosolic pyruvate kinase monomers PKM1 and PKM2 (22, 23). Subsequently, reports identified a 55 kDa protein displaying disulfide-isomerase activity (24) in addition to a high affinity for T3 and estrogen (24, 25). Several follow up reports characterized this protein as protein disulfide isomerase (PDIA1 or P4HB), the first folding catalyst discovered, as mainly responsible for mediating oxidative protein folding in the endoplasmic reticulum (25–27). As a dithiol-disulfide oxidoreductase, PDIA1 reduces, oxidizes and isomerizes disulfide bonds, helping to maintain a healthy calcium-rich oxidative environment in the endoplasmic reticulum lumen (26, 28). Another important function for this enzyme is its chaperone activity, which has been well-studied both *in vitro* and *in vivo* (29, 30).

PDIA1 is the founding member of a family containing more than 20 members and it comprises four thioredoxin domains: a-b-b′-a′. The “a” domains exhibit redox catalytic WCGHC motifs, or the so-called “redox cysteines,” which are essential for PDIA1 function as an oxidoreductase. The thioredoxin fold structure found in both “b” domains present no redox cysteines but is enriched in hydrophobic residues involved in substrate recognition and binding. Connecting b′ and a′ domains, there is a 19-residue short interdomain region, named x-linker, while at the C-terminus region there is a highly acidic extension, involved in calcium binding and a KDEL motif (endoplasmic reticulum retrieval motif) (28).

Among all pathophysiology involving PDIA1, it is important to mention its role in the following: (i) neurodegenerative and protein misfolding-associated diseases; (ii) cancers, involving events such as cell migration and metastasis; (iii) endoplasmic reticulum stress; (iv) cytosolic retrotranslocation of un/misfolded proteins for proteasome-mediated degradation; (v) autoimmune processes, such as rheumatic heart disease; and (vi) as a bisphenol-A-binding protein in rat brains [for more information see review (28, 31)].

Considering the binding of estrogen hormone to PDIA1, it was shown that PDIA1 colocalized with estrogen hormone receptor α (ERα) in MCF-7 cell nuclei, thereby altering ERα conformation and enhancing the ERα-ERE interaction. Consequently, PDIA1 was able to mediate changes in gene expression regulated by ERα (32). In addition, the overexpression of PDIA1 in GH3 cells suppressed growth hormone (GH) mRNA expression and GH release, suggesting that PDIA1 modulates T3-induced gene expression (33). Follow-up studies clarified that PDIA1 plays a role in this gene regulation mechanism, as it modulates the redox state of Ref-1 (redox factor-1), which is responsible for changing the TR redox state and controlling GH gene expression (34).

Here, we found PDIA1 as a new partner of TRs. Through a yeast two-hybrid screening assay, we found that PDIA1 interacted with TRβ. Furthermore, we explored whether PDIA1 was able to directly bind to TRs, whether this interaction is guided by a specific TR isoform, whether the presence of T3 was relevant for this association and, most importantly, whether the TR:PDIA1 complex might influence TR-dependent gene regulation. Interestingly, we found that, in addition to interacting with TRs, PDIA1 plays a functional role in their modulation, thus altering target gene expression through a mechanism that may involve PDIA1 thiol reductase/isomerase activity.

## Materials and Methods

### Plasmid Constructs

#### Yeast Two-Hybrid Assays

Oligonucleotides were designed to amplify and sub-clone the cDNAs encoding the amino acid sequence of the full length human TRβ in the pBTM116 vector.

#### Mammalian Cells Assays

The coding sequence of full-length human Flag-TRα1 and human Flag-TRβ1 were subcloned into lentiviral vector LV-IG in XbaI+NheI sites, constructed by LVV Facility (Viral Vectors Lab inside LNBio), which consists in bicistronic IRES-GFP vector to quickly identify cells expressing the protein of interest by fluorescence microscopy. A plasmid containing flag tagged eGFP gene (eGFP-flag) was used as control in immunoprecipitation experiments.

#### Reporter Gene Luciferase Assays

Constructions containing Response Elements for TR (DR-4, F2, AP-1) were cloned as transcription regulators of Firefly Luciferase gene. A plasmid containing *Renilla* Luciferase (pRL) was used as internal control of transfection for data normalization. Other plasmids used contain the human gene of PDIA1 full length (pcDNA3.1- PDIA1); TRα-1 (pcDNA3.1-TRα1), and TRβ-1 (pcDNA3.1-TRβ1) full length.

#### Protein Expression in E. coli BL21 (DE3)

All genes were cloned in pET28a(+) expression vector, hTRα full length, hTRβΔAB (amino acids 102–461), and hPDIA1 full length, kindly donated by Dr. Francisco Laurindo's Research Group.

#### Single Point Mutants

TRβ-C294A or TRβ-C298A mutated plasmids were constructed by site-directed mutagenesis, following the protocol established in the QuikChange II Site-Directed Mutagenesis kit (Agilent Technologies). Primers for mutagenesis experiments were designed using the QuickChange® Primer Design Program (Agilent Technologies).

### Yeast Two-Hybrid Screening (y2h)

The Screening of TRβ against HeLa Library was performed in *Saccharomyces cerevisiae* strain L40 [trp-25, his3Δ200, leu2-3, ade2, LYS2::(lexAop) 4-HIS3, URA3::(lexAop)8lac GAL4], which contains the heterologous genes HIS3 and lacZ, according to a previous published study (35). The pBTMK-TRβ vector was used to express the full-length human TRβ, and pBTM116K-empty vector was used as a control. The autonomous activation of HIS3 gene was tested by co-transformation of yeast cells with pBTMK-TRβ and pACT2-empty vector (control), grown in minimal medium plates (without tryptophan, leucine, histidine) containing 5 mM 3-amino-1,2,4-triazole (3-AT). Also, we performed Beta-Galactosidase Test, in which colonies expressing Gal4 gene becomes blue, meaning that transcription factor was reconstituted by protein interaction. The y2h screenings were performed against HeLa cDNA library, cloned in pACT2 vector, expressing GAL4 activation domain (Matchmaker System, Clontech). After discovering the interaction partners, we performed co-transformation to confirm each interaction.

### Cell Culture

293T (ATCC® CRL-3216™) and Hep G2 (ATCC® HB-8065™) cells were grown in Dulbecco's modified Eagle's medium (DMEM) supplemented with 10% fetal bovine serum (FBS), 100 U/mL of penicillin, 0.1 g/L of streptomycin and 4 mmol/L glutamine, under 95% air and 5% CO_2_ at 37°C.

### Co-immunoprecipitation TR-PDIA1

To validate the TR-PDIA1 interaction, we performed co-immunoprecipitation (Co-IP) experiments in 293T cell line expressing flag-TRs. Cells were grown until 70% confluence and plated in 100 mm petri dishes. Next day, cells were transfected with Flag-TRα1 or Flag-TRβ1 or empty vector. Four hours later cells were treated with T3 or DMSO (negative control) for 36 h. After treatment, cells were collected and lysed in RIPA buffer. The lysates were cleared by centrifugation (9,000 × g, 20 min, 4°C) and incubated overnight with Mouse anti-FLAG (M2 affinity gel, Sigma) or mouse anti-PDIA1 (R&D, #MAB4236). The beads were gently washed by centrifugation 3 times in Tris-buffered saline (TBS) pH 7.5 and the complexes were eluted by boiling the samples before running the SDS-Page gel. In the flag-TRs immunoprecipitation, through western blot (WB), we searched for PDIA1, using anti-PDIA1 (mouse anti human PDIA1, R&D #MAB4236). Aiming to confirm the interaction, we also immunoprecipitated PDIA1 and searched for the transient transfected flag-tagged TRs through WB, using specific antibodies (rabbit anti-DYKDDDDK Tag—Cell Signaling #2044; mouse anti-flag—Sigma Aldrich #A8592; mouse anti-TRα–R&D, PP-H2804-00; mouse anti-TRβ–R&D, PP-H3825A-00). Bands were quantified through Image Studio^TM^ Lite (Li-Cor).

### Expression and Purification of TRs

hPDIA1 full length, hTRα full length and hTRβΔAB (amino acids 102–461) were expressed using pET28a(+) expression vector in BL21(DE3) *Escherichia coli* cells. Bacteria harboring the expression plasmid were grown in LB medium at 22°C, and protein expression was induced with 0.5 mmolL^−1^ IPTG (16 h). Cells were harvested by centrifugation (4,000 × g, 15 min, 4°C). TRα and PDIA1 pellet were suspended in BUFFER A (20 mmolL^−1^ Phosphate Buffer pH 7.5, 300 mmolL^−1^ NaCl, 5% glycerol), and TRβΔAB in BUFFER B (20 mmolL^−1^ Hepes pH 8, 300 mmolL^−1^ NaCl, 5% glycerol). In both buffers, 2 mmolL^−1^ of β-mercaptoethanol, 100 mmolL^−1^ PMSF and lysozyme were added to the suspension. Cell extracts were sonicated and centrifuged (30,000 × g, 60 min, 4°C) in Avanti J26xPT centrifuge (Beckman Coulter, rotor JA-25-50) for clarification. Proteins extracts were incubated with Talon resin (Clontech) (1 L culture/1 mL resin) and were eluted with Buffers A or B with 300 mmolL^−1^ imidazole and 2 mmolL^−1^ of β-mercaptoethanol addition. Purity of samples was evaluated by SDS-PAGE. In addition, Size-Exclusion Chromatography (SEC) performed with Superdex 75 16/600 (GE Healthcare) column was used to improve purification of the proteins hTRα full length, TRβΔAB, and hPDIA1 full length, obtained in affinity purification. The column was previously equilibrated with modified buffers A or B, with 150 mmolL^−1^ NaCl each. After that, proteins (2 mL, at ~8 mg/mL) were injected into the column and purification was performed at a flow rate of 0.3 mL/min. The isocratic elution was made with 1.5 CV. Fractions containing pure proteins were gathered and concentrated to be used in the next assays.

### Analytical Gel Filtration (GFSEC-GF)

To analyze the radii from the proteins alone and in complex we utilized Superdex 200 HR 10/300 size exclusion column (1 × 30 cm; GE Healthcare). The column was equilibrated with modified buffers A or B (150 mmolL^−1^ NaCl, 2 mM DTT added) at a flow rate of 0.5 mL/min and standardized with the gel filtration calibration kits (GE Healthcare), applied in a volume of 100 μL. Ferritin, Aldolase, Conalbumin, Ovalbumin, Carbonic Anhydrase, Ribonuclease and Aprotinin with hydrodynamic radii of 6.1, 4.8, 3.6, 3.0, 2.0, 1.6, and 1.3 nm, respectively, were used as calibration standards as recommended by manufacturers. The elution volumes of these proteins were used to calculate estimated hydrodynamic radii as described in $(36). After calibration, following samples were injected and analyzed: TRα and TRα:PDIA1 (500 μL at ~0.75 mg/mL); PDIA1 (100 μL at ~2.5 mg/mL); TRβ and TRβ:PDIA1 (100 μL at ~2.5 mg/mL). All curves profiles were analyzed in GraphPad Software.

### Dynamic Light Scattering (DLS)

Pure proteins (hPDIA1 full, hTRα full, hTRβΔAB), or in complex (TRα:PDIA1 and TRβ:PDIA1), at ~1 mg/mL, were analyzed in Dynamic light scattering (DLS) measurements. Those were conducted using the ZetaSizer NanoZS90 (Malvern) equipped with a 632.8-nm He-Ne laser and operating at an angle of 90°. For each sample, one measurement corresponds to 15–100 acquisitions (according to sample concentration) of 10 s, at 15°C with an automatic attenuator (Attenuation 11). The intensity size distribution, the Z-average radius (Z-ave) and the polydispersity index (PdI) were obtained from the autocorrelation function using the “Protein Analysis mode” for the protein sample. The software used to collect and analyze the data was the ZetaSizer Software version 7.11 from Malvern.

### Fluorescence Anisotropy of TR:PDIA1 Complex

The affinities of TRαfull and TRβDL for PDIA1 were measured by fluorescence anisotropy. TRαfull and TRβDL were previously labeled with fluorescein isothiocyanate (FITC) as previously described (37–39). Purified PDIA1 were titrated from 0.012 to 25 μmol/L into 50 nmol/L TR(α or β)-FITC solution, with and without triiodothyronine (T3), in assay buffer (20 mM Sodium Phosphate pH 7.4, 150 mM NaCl, 5 mM MgCl_2_ and 1 mM DTT) in a 384-plate (Greiner, non-binding). For the experiments performed in the presence of T3, TRα, and TRβ were previously incubated with 3 times molar excess of ligand, for 30 min, at 5°C. After mounting the plate series, specimens were left under incubation at 5°C for 20 min before the measurement. Fluorescence polarization was measured in CLARIOstar (BMG Labtech), Fluorescence Polarization—Endpoint was used as the detection mode, as well as, a 485 nm excitation filter, 565 nm longpass filter and a 540 nm emission filter, at 20°C. Focus and Gain adjustments for both channels were set as recommended by manufacturers and all fluorescence experiments were performed at least in triplicate. Acquired data were analyzed using Mars Software and graphically edited in Origin software (version 8.0; OriginLab Corp), which applies the sigmoidal Hill1 model for fitting curves to determine the affinity constant (K_d_) and Hill coefficient values.

In addition, in order to evaluate if oxidized or reduced PDIA1 presented different affinities for TRα and TRβ, we first incubated PDIA1 with 0.5 mM H_2_O_2_ or with 0.5 mM DTT, for 30 min, to oxidize or reduced PDIA1 samples, respectively. Immediately after this incubation, PDIA1 were titrated from 0.012 to 25 μmol/L into 50 nmol/L TR(α or β)-FITC solution, with and without triiodothyronine (T3), in assay buffer (20 mM Sodium Phosphate pH 7.4, 150 mM NaCl, 5 mM MgCl_2_) in a 384-plate (Greiner, non-binding) and Fluorescence polarization was measured as previously described. We also aimed to oxidize TRs to perform the same experiments, however oxidized TRs are not stable and forms aggregates (data evaluated by DLS, not shown) and, for this reason we just evaluated PDIA1 redox states in TR binding.

### Reporter Gene Assays

293T cells were trypsinized, resuspended in DMEM, plated in 24-well plates (density of 1.1 × 10^5^ cells/well), and incubated with the following plasmids: pBlueScript (used as empty DNA to equilibrate DNA quantity); pRL (which contains *Renilla reniformis* luciferase) used as the transfection control; F2-Luc (plasmid that contains positive response element for TR followed by firefly luciferase reporter gene); DR-4Luc (plasmid that contains positive response element for TR followed by firefly luc); AP-1Luc (plasmid that contains negative response element for TR followed by firefly luc); PDIA1 (plasmid with PDIA1 full gene); TRα (plasmid with TRα full gene); TRβ (plasmid with TRβ full gene); TRβ-C294A (plasmid with TRβ C294A mutant full gene) or TRβ-C298A (plasmid with TRβ C298A mutant full gene). Plasmids were mixed with Lipofectamine® 2000 (Invitrogen) in a ratio of 1.5 μg of DNA to 2 μL of Lipofectamine, at room temperature, for 20 min before addition to the cells. Hormone T3 (obtained from Sigma-Aldrich) was added at 1 μM to the culture medium 4 h after the transfection. The cell monolayer was harvested 48 h later with lysis buffer (Dual Luciferase® Report Assay System; Promega) according to the manufacturer's instructions. Luciferase activity was determined using Dual Luciferase® Reporter Assay System (Promega) and measured in GloMax-Multi+ detection system (Promega). The *R. reniformis* luciferase activity was measured using the same cell lysate and used as an internal control. Transfection of the Response elements (DR4, F2, AP1) together with pBlueScript and treatments with T3 or DMSO were performed as a control of each reporter activation assay. In control all luciferase signals are due to endogenous activation of any endogenous NR. The activation values for TRs (wild type and mutants) and PDIA1 were obtained by normalization with this control. Statistical Analysis with One or Two-way ANOVA ^*^p < 0.05, ^**^p < 0.01, ^***^p < 0.001.

### Knockdown PDIA1 Followed by qRT-PCR of T3 Target Genes

SMARTpool ON-TARGETplusP4HB small interfering RNA (siRNA; number L-003690-00-0010) or non-targeting control (Dharmacon, GE Healthcare) was transfected into HepG2-TR using Dharmafect cell culture reagent 4 (Dharmacon, GE Healthcare) according to manufacturer's instructions. Following transfection, cells were cultured in medium for 48 h and then replicated into new plates. After 24 h of growth, cells were transfected again with siRNA, to increase the efficiency of the knockdown procedure; and 4 h later, T3/DMSO were added to media. Cells were collected 24 h later and all reactions were performed in triplicate.

We confirmed the knockdown through WB, so we compared the expression of PDIA1 (mouse anti human PDIA1, R&D #MAB4236) with the control beta-Actin (mouse anti-beta Actin—ABcam #ab6276). Total RNA was extracted from cells using Qiazol Lysis Reagent (Invitrogen), and purified by RNeasy Mini kit (Qiagen) following manufacturer's instructions. Reverse transcription reactions were performed using 1 mg of total RNA with an iScript cDNA Synthesis kit (Bio-Rad). Total RNA concentrations were measured using NanoDrop ND-1000 spectrophotometer. Real-time qPCR was performed in Roche LightCycler 480 RT PCR Instrument using SYBR Green Mastermix (Roche). The sequences of the primers are listed in Supplementary Table 1. The data were collected and analyzed using the comparative threshold cycle method. Experiments were performed at least three times, and the standard error was calculated using the Prism curve-fitting program (GraphPad Software, version 3.03; GraphPad). Amplification curves were evaluated by the comparative Ct analyses.

### Fluorescence Anisotropy of Coactivator Peptide SRC1

The affinities of the complex TR+T3:PDIA1 for coactivator peptide SRC1 were measured by titration assays using fluorescence anisotropy, as previously described (38). The labeled coregulator peptide SRC-1 (Fluorescein-SRC1–1 Coactivator Peptide 3 FITC-KYSQTSHKLVQLLTTTAEQQL) was obtained from Life-technologies. In control experiment, we titrated TR+T3 from 1 to 7,000 nM in SRC1 at 50 nM. After that, we titrated the complex TR+T3:PDIA1 with the same concentrations gradient in SRC1. Protein complex stocks were diluted in the same buffer A or B. All fluorescence curves were fit as described (37, 38), using Origin software (version 8.0, Origin- Lab Corporation), which applies the Levenberg-Marquardt algorithm for fitting curves to non-linear equations, to determine the Kd and Hill coefficient values. All fluorescence experiments were performed in triplicate.

### Molecular Docking

Molecular docking experiments aimed to investigate structurally the interaction between PDIA1 and TRα or TRβ. Crystal structures of human TRα [PDB ID: 2H77, (40)] and TRβ [PDB ID: 3GWS, (40)] were obtained from Protein Data Bank (PDB, http://www.rcsb.org/pdb/). Full length human PDIA1 structure was modeled based on the oxidized form of yeast PDIA1 [PDB ID: 2B5E, (41)] and using the software YASARA (42). Docking experiments were performed, without any distance constraints, using the web server for protein-protein interaction prediction ClusPro (43). Additionally, the energy of the best predicted models was minimized using the software YASARA.

## Results

### PDIA1 Is a New TR Partner

**Figure 1:**
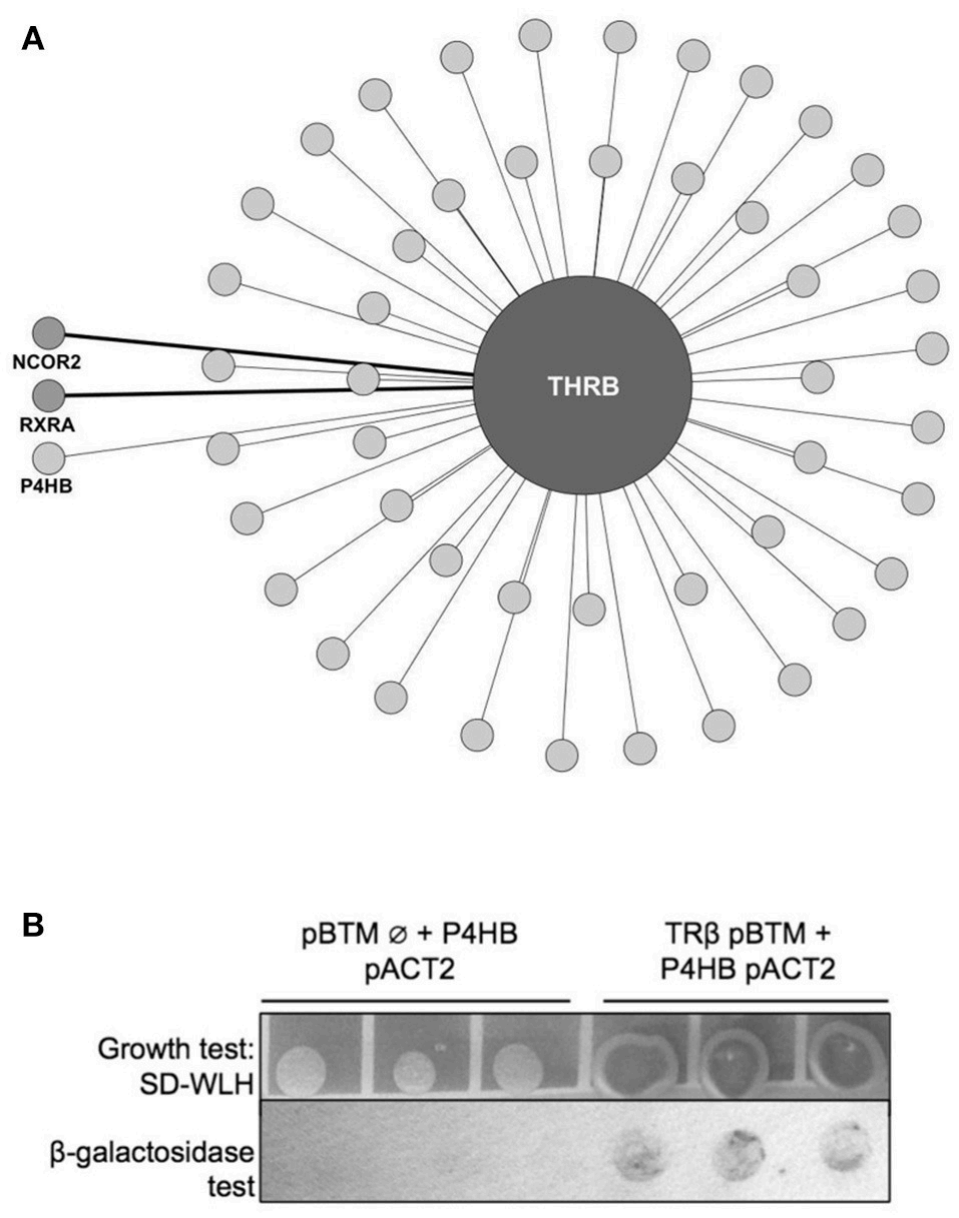
TRβ yeast two-hybrid (y2h) screening. **(A)** Network created with IIS (Integrated Interactome System) platform and built in Cytoscape 3.3.0 software. Y2H experiments identified 50 preys, among them, light-gray circles represent the new interactors found for TRβ (48 in total), and medium-gray, detached from circle, the already-known TR partners NCOR2 (Nuclear Receptor Corepressor 2) and RXRA (Retinoid X-Receptor α). In light-gray, also detached from circle, we show the new TRβ partner found, Protein Disulfide Isomerase (PDIA1, also known as Prolyl 4-Hydroxylase Subunit Beta—P4HB). **(B)** Confirmation of PDIA1 and TRβ interaction found in Y2H assay. The TRβ and PDIA1 interaction reconstructed cell transcription factors machinery and transcribed genes that allows colonies to grow, we observe this in dark-grey colonies. Moreover, this interaction produced blue products in the β-galactosidase assay. Negative control with an empty vector (pBTM Ø) had no growth and in β-galactosidase assay, no blue color.

Aiming to find new partners for TR, we first performed a yeast two-hybrid screen (y2h) using TRβ as the bait (Figure 1A). The TRβ interactome map (Figure 1A) shows 48 new and 2 known protein partners. These 2 interactors for TRβ are NCor2 and RXRα, which have already been described as TRβ partners (detached from the circle in Figure 1A, medium-gray colored), which brings higher confidence to our data (3, 44–46). Among all 48 new potential interactors found with this assay (light-gray colored, Figure 1A), we found P4HB (the gene coding for PDIA1). This protein was considered particularly important since it is also able to bind T3 hormone and to regulate a subset of TR genes (34). Subsequently, we decided to perform follow-up interaction confirmation assays (β-Galactosidase and growth tests). These assays confirmed a TRβ:PDIA1 interaction (Figure 1B), as the cotransformation of yeast with both genes, TRβ and PDIA1, presented better growth in comparison with control cotransformants (empty vector and PDIA1 gene). Thus, a TRβ:PDIA1 interaction was confirmed in yeast.

### PDIA1 Binds TRα and the TRβ Interactor in 293T Cells in the Absence or Presence of T3

**Figure 2:**
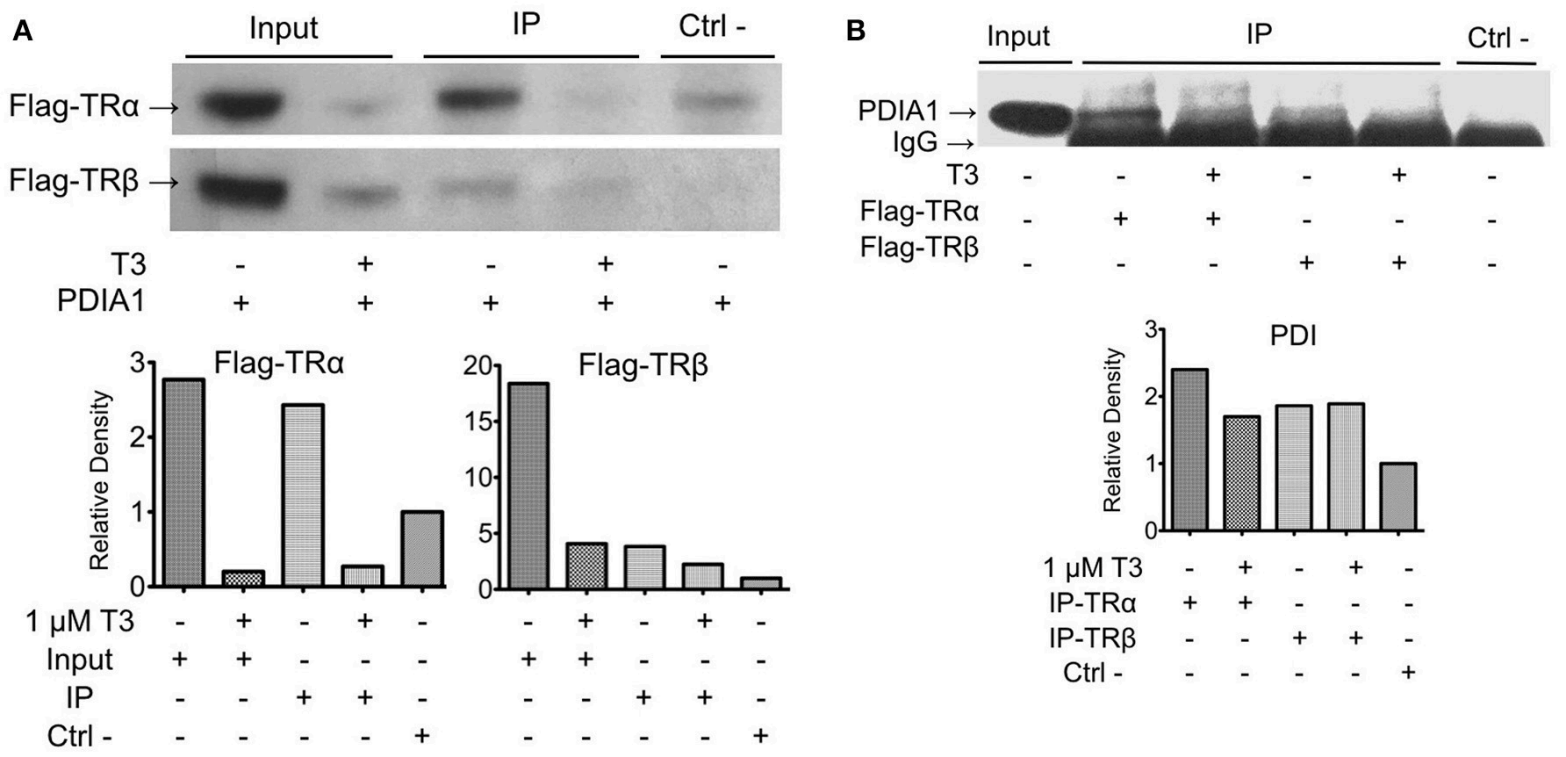
Co-immunoprecipitation of TR:PDIA1 in 293T cells shows the interaction for both isoforms with or without T3 presence. **(A)** Immunoprecipitation of PDIA1 followed by Western Blot anti-flag, revealing Co-IP of PDIA1 and flag-TRs. The treatment with T3 (1 μM) decreases TR expression in 293T cell (input bands are thinner) reducing protein available to be immunoprecipitated. The interactions of TRs with PDIA1 were confirmed according to the quantification of bands (presented in the graphs below the WB). **(B)** Immunoprecipitation of flag tagged TRs (+ or –T3), and western blot anti-PDIA1. Control experiment WB anti-flag is presented in Supplementary Figure 2A, together with a confirmation experiment (Supplementary Figure 2B). Although WB shows strong IgG band, it is still possible to observe the presence of PDIA1 in all conditions, co-immunoprecipitated with both isoforms of TR. The quantification of bands are presented in graphs below WB, where all signals were normalized to the control signal (relative Density). 293T cell extracts without T3 and without transfection of flag-TRs were used on this experiment as negative control. Co-IP and WB experiments were performed more than once and here we show a representative image.

Once we found and confirmed the PDIA1 interaction in TRβ y2h, we tested whether this interaction was modified by the presence/absence of ligands, or if it was selective for any TR isoforms (TRα and TRβ) in 293T cells. To do that, first, we confirmed the exogenous expression and activity of both TRs in 293T cells in reporter gene assays (Supplementary Figure 1). Band quantification showed some decrease in TR expression after T3 treatment, which was previously reported (17, 47) and was not relevant for our assay. In addition, the flag-TR reporter gene assay presented a considerable response to hormones (Supplementary Figure 1). After confirming these parameters, co-IP experiments for TRs and PDIA1 (Figure 2) showed that when PDIA1 was immunoprecipitated, more TRs were coimmunoprecipitated, mainly in the absence of ligands (Figure 2A), which was true for both TR isoforms, presenting additional evidence of a TR:PDIA1 interaction inside cells. Additionally, the inverse coimmunoprecipitation experiment was also performed, and the immunoprecipitated TRs were able to bring PDIA1, reinforcing the TR:PDIA1 interaction (Figure 2B). Although we observed IgG bands below PDI bands in the WB (Figure 2B), the quantification band software (illustrated in the graph below each co-IP western blot, Figure 2) helped us to analyze the presence or absence of antibody signals in each co-IP assay. Based on all the above tests, we confirmed one interesting, new protein partner for TR; moreover, we showed that this weak interaction, although visible through image analysis, is not responsive to T3 or selective to any TR isoform.

### Biophysical Characterization of TR:PDIA1 Complexes

Biophysical assays were performed to confirm and characterize the direct interaction between TR:PDIA1 *in vitro*, aiming to gain more information about this complex formation and to address conditions in which this interaction happens.

First, SEC-GF experiments showed that TR:PDIA1 behaves as a complex, presenting an elution profile consistent with that of a bigger molecule than the isolated proteins. We also observed the presence of proteins alone and in complex through SDS-Page gels (data not shown) in each SEC and GF run, and bands from TR and PDI were observed in the complex lane. The calculated hydrodynamic radius of TR:PDIA1 complex in this assay was 4.0 nm, which was higher than the calculated R_h_ for TRα (2.8 nm), TRβ (2.9 nm), and PDIA1 (3.2 nm) alone. These R_h_ indicated that TRα, TRβ, and PDIA1 behave as monomers in solution and suggest that the TR:PDIA1 complex is formed by one TR and one PDIA1 monomer (Supplementary Figure 3, Supplementary Table 2).

Additionally, we subjected the TRs, PDIA1, and TRs:PDIA1 complexes to DLS experiments. The light scattering experiments revealed that TRs and PDIA1 behave as monomers, with a R_h_ more similar to those described in the literature [PDIA1 R_h_ = 3.3 nm (48) and TRs R_h_ monomer = 3.6 nm (36)], while TRs:PDIA1 complexes exhibited a R_h_ = 4.2 nm. These differences in the absolute values may be intrinsic of technical approaches; however, in all cases, our results showed that TRs and PDIA1 behave as monomers in solution and their mixture behaves as larger units in SEC-GF and in DLS assays, which is a strong evidence of stable complex formation (Supplementary Table 2).

### The Binding of PDIA1 to TRs *in vitro* Is Also Independent of T3

To access more information about complex formations, as well as isoform and ligand preferences, we performed fluorescence anisotropy assays to measure affinities between PDIA1 and TRα or TRβ in the presence or absence of T3. Our results showed that affinity constant (K_d_) values obtained from the binding curves (**Figure 4**) were ~2 μM for both TR isoforms in a reduced environment. When PDIA1 was titrated in TRα, we obtained K_d_ was 2.05 ± 0.24 μM in the absence of T3, and 1.97 ± 0.18 μM in the presence of T3. In addition, when PDIA1 was titrated in TRβ, the K_d_ were 2.71 ± 0.27 μM and 2.81 ± 0.42 μM, respectively, without and with T3. Thus, we unambiguously confirmed that, in this assay condition, the presence of T3 was not able to induce any significant difference in the affinities between the TRs and PDIA1. Most importantly, these assays also showed that PDIA1 and TRs were able to interact with each other *in vitro*, displaying an affinity of ~2.0 μM, without discrimination between TR isoforms.

### PDIA1 Changes the Transcriptional Activity of TRs in 293T Cells

As TRs are nuclear receptors that regulate the expression of several genes and we were able to identify a direct interaction between PDIA1 and TRs, we investigated, in 293T cells, whether PDIA1 could interfere in the transcriptional activation of TRs. Using a reporter gene assay, we demonstrated, as expected (49, 50), an increase in transcriptional activity for both TRs in the presence of T3 at the F2 positive hormone response element (HRE) (**Figures 4A,B**). Moreover, we observed that in this HRE, when we added only PDIA1 to the system, the basal activation of TRα was increased 4-fold (**Figure 4A**). Additionally, in the presence of T3, which *per se* increased basal TRα activation 9-fold, the TRα:PDIA1 activation was increased by an additional 3-fold, for a 25-fold increase over the basal TRα activity (**Figure 4A**). In parallel, for TRβ, in the F2-HRE system, PDIA1 incubation increased basal activity by 2-fold (**Figure 4B**) independently of the presence of T3. We also observed that T3 increased TRβ activity by 50-fold, and PDIA1 addition further increased this activation 2-fold, reaching a total of 115-fold of the basal TRβ activity (**Figure 4B**). In this way, in an activation context, PDI increased TRα and TRβ activation.

We also observed the previously reported (51) TR gene repression by T3 at the AP1 negative response element (52) (**Figures 4C,D**). Interestingly, our results showed that for the TRα assay (**Figure 4C**), PDIA1 incubation was able to decrease basal gene expression in both with and without T3. Additionally, T3 and PDIA1 together were able to reduce the basal TRα activation by 50%. However, it is important to mention that, in the absence of TRα, PDIA1 showed basal repression of AP1, suggesting that PDIA1 action in AP1 promoter repression may be independent of TRα.

Surprisingly, for β isoforms, in this same AP1-HRE (**Figure 4D**), we observed a different scenario. When PDIA1 was added to the system in the absence of T3, basal activation increased to 1.5-fold, while in the presence of T3, basal TRβ activation decreased ~60%. In addition, PDIA1 and T3 together sustained the repression of basal activation, but at lower levels (~40%). In the latter case, PDIA1 and T3 appeared to work in opposing directions depending on the TR isoform, and PDIA1 together with TRβ appears to modify coregulator binding.

In summary, based on these experiments, we obtained two important findings. First, PDIA1 presence alone is sufficient to perturb TR gene regulation, depending on the promoter region observed. Specifically, our experiments showed that PDIA1 proportionally enhances TR activation in positive HREs, independently of the presence of T3. Second, we also showed that PDIA1 may regulate each TR isoform in a different and specific way concerning negative HREs, suggesting that it modulates TRβ repression in a stronger way than does the α isoform.

### PDIA1 Knockdown Affects TR-Regulated Gene Expression

As PDIA1 was able to affect the transcriptional activity of both TRs, we performed PDIA1 knockdown to investigate whether PDIA1 effects sustain the expression of some well-described genes regulated by T3 (16). First, we observed that siRNA transfection in Hep-G2 cells expressing TRα or TRβ strongly reduced PDIA1 levels (**Figures 5A,B** and Supplementary Figure 4). Moreover, siRNA-mediated PDIA1 knockdown was confirmed at both mRNA and protein expression levels (**Figures 5A,B**, Supplementary Figure 4). Additionally, we further investigated the changes in Furin, Myh6 and Hif2a gene expression, known to be under strict T3 regulation (16) (**Figure 5C**).

Analysis of Hif2a gene expression (**Figure 5C**) showed increased gene transcription after PDIA1 knockdown for both TR isoforms, with a more evident increase in the presence of T3. Accordingly, Myh6 (**Figure 5C**) also behaved as a gene negatively regulated by PDIA1, given its increased expression after PDIA1 silencing. Interestingly, Furin gene expression (**Figure 5C**) showed no significant change after PDIA1 silencing, suggesting that PDIA1 is not essential for this specific gene regulation.

Given these results, we showed that PDIA1 may exert functional roles as a repressor of TR-regulated gene transcription, but apparently in a way restricted to specific genes, which should be investigated further.

### PDIA1 Changes the Recruitment of CoA by TR

After characterizing the TR:PDIA1 interaction and showing that PDIA1 affects the activity of TR, we next investigated whether TR:PDIA1 complex formation alters the recruitment of TR coactivators. For that, we performed a fluorescence anisotropy assay to check the binding of TRs+T3:PDIA1 to the SRC1 peptide.

Prior to the study of the TR:PDIA1 complex, we addressed the binding of TRs to SRC1 as a control (Figures 6A,B). Given the K_d_s found for SRC1:TRα+T3 (0.29 μM) and SRC1:TRβ+T3 (0.68 μM), we assumed that both isoforms have a very high affinities for SRC1 peptides (Table 1). Following these K_d_ determinations, we showed that PDIA1 incubation changes the affinities of TRα or TRβ in different ways. The affinity for coactivators was decreased with TRα but increased with TRβ (TRα from 0.29 ± 0.04 μM to 0.7 ± 0.1 μM, and TRβ from 0.68 ± 0.06 μM to 0.39 ± 0.06 μM, Table 1). These results suggested that PDIA1 is not competing for coactivator recruitment because the complexes were able to bind to CoA peptide with K_d_s in the same order of magnitude as in the absence of PDIA1. Additionally, we propose that PDIA1 provokes conformational changes that might improve TRβ:SRC1 binding and impair TRα:SRC1 binding.

**Table 1:** Measured dissociation constants (Kd, uM) of SRC1 binding to TR+T3 isoforms in the presence and absence of PDIA1.

	**SRC1 titrated in TRα+T3**		**SRC1 titrated in TRβ+T3**	
	**–PDIA1**	**+PDIA1**	**–PDIA1**	**+PDIA1**
K_d_, μM	0.29 ± 0.04	0.70 ± 0.10	0.68 ± 0.06	0.39 ± 0.06

### Computational Modeling for the Hypothetical Surface of Interaction Between PDIA1 and TR

Aiming to understand the structural mechanisms of interactions between PDIA1 and TRα or TRβ, we performed molecular docking experiments. Using the web-based server ClusPro, we obtained a total of 111 clustered complexes for each TR isotype and selected the centers of the most populated clusters of low-energy structures as putative models (43).

The docking of the TRα:PDIA1 complex resulted in two equally most likely conformational clusters. The central structure of the first cluster proposes that TRα (green) interacts with PDIA1 (gray) fitting into the “boat” arrangement formed by the four domains of PDIA1 (**Figure 7A**, Model 1). One of them, the b′ domain, accommodates one segment of the hinge region through a hydrophobic contact with the L154 residue of TRα. Additionally, PDIA1 a and PDIA1 a′ domains make direct contacts with helices H10 and H11, and H1, H2 and H10 of TRα, respectively. The C-terminal helix H12, in turn, does not participate in the TRα:PDIA1 interface. In addition, an equally probably second model was suggested (model 2). This alternative also shows TRα buried into the PDIA1 domains, but differently from the first model, and TRα interacts in an alternative orientation (**Figure 7B**). In this model, the hinge does not contact PDIA1, but a more extensive interface is formed among TRα, the PDIA1 a domain (H1, H3, H4, H5, and H12), and the PDIA1 a' domain (H8, H10, and H11) was observed. Furthermore, residue E403 of H12 (TRαcan make direct contact with the residue R97 from PDIA1 in this model.

Interestingly, docking of TRβ to PDIA1 resulted in one significantly more populated cluster, with TRβ presented in the same orientation as that of TRα model 2 (**Figure 7C**). In addition to the already described contacts for TRα model 2, this orientation of the TRβ:PDIA1 complex allows pairing of two cysteines from TRβ (C294 and C298) with the catalytic cysteines of PDIA1 (C53 and C56). As TRβ and TRα model 2 are in the same orientation in relation to PDIA1 (**Figure 7D**), it would be expected that a similar pairing of cysteines was also observed. However, only TRβ has two cysteines to be paired, while TRα has just one cysteine (C244) in the correspondent region. Moreover, TRα C244 is located further away from PDIA1 catalytic cysteines than TRβ C294 or C298 (**Figure 7**).

### Site-Directed Mutagenesis of TRβ-Cysteines

According to the proximity between the catalytic cysteines of PDIA1 and the cysteines of TR presented in our docking model, we evaluated whether PDIA1 disulfide isomerase can affect TR activity by forming disulfide bonds at its paired cysteines. For this, we performed transactivation assays of mutated TRβ (TRβ-C294A and TRβ-C298A) in F2-response elements. Based on this, we expected that mutation of one cysteine from TRβ would lead to loss of its activity.

The mutations did not reduce overall activity of TRβ, which maintained its transactivation function in the presence of T3 (**Figure 8A**). Surprisingly, incubation of PDIA1 in the TRβ mutant transactivation assay (**Figure 8B**) led to increased TRβ-C294A and TRβ-C298A basal activation by ~3- to 4-fold. On the other hand, in the presence of T3, PDIA1 promoted further minor reductions of TRβ-C294A and TRβ-C298A activation in comparison with wt TRβ (86-fold for C294A, 89-fold for C298A and 115-fold for wt TRβ). From this experiment, we concluded that the absence of one cysteine from TRβ was not capable of totally preventing PDIA1-mediated regulation of this receptor, but the decrease in TR+T3 activation was in line with TRα wt behavior, which presented lower activation in comparison to TRβ wt. Thus, we suggest that the PDIA1 makes some influence in TR activation, more specifically over TRβ activation, which was made more significant by the presence of two cysteines (wt TRβ) in comparison with just one cysteine (wt TRα or TRβ-C294A and TRβ-C298A).

### Changes in the PDIA1 Redox State Modifies the Affinities for TRβ

To investigate whether the PDIA1 redox state might affect the binding of PDIA1 to TR, we determined its affinity for TR under a reducing (in presence of DTT) or oxidizing (in presence of H_2_O_2_) condition (Table 2, Figure 9). For TRα, the PDIA1 redox state did not significantly change the affinity between both proteins (Kd_TRα−*PDIA*1*Oxi*_ = 7.9 ± 1.8 μM and Kd_TRα−*PDIA*1*Red*_ = 10.7 ± 1.3 μM), but the Kds were slightly higher than the ones determined in the experiment done in the reduced environment (Kd ~2 μM) (Table 2, **Figure 9**). This Kd difference may be explained by differences in experimental conditions since the first experiment used a reductive buffer, while the second was performed in conditions that did not favor reduction or oxidation, but the affinities are considered similar since they are in the same magnitude order. In other words, this experiment shows that oxidation or reduction of PDIA1 did not affect binding to TRα. On the other hand, for TRβ, PDIA1 oxidation disrupts TRβ binding while PDIA1 reduction favors TRβ association (Kd_TRβ−*PDIA*1*Oxi*_ >> 102 ± 264 μM and Kd_TRβ−*PDIA*1*Red*_ = 4.54 ± 0.45 uM), indicating that the PDI redox state might be involved in TRβ recognition (Table 2, **Figure 9**). Moreover, our results suggested that a reduced environment favors PDIA1-TR association, which is more evident for a TRβ isoform that has two cysteines, probably interacting with PDIA1.

**Table 2:** Measured dissociation constants (Kd, uM) of reduced and oxidized PDIA1 (PDI _Red_ or PDI_Oxi_ binding to TR isoforms.

	**PDIA1 titrated in TRα**		**PDIA1 titrated in TRβ**	
	**Oxi**	**Red**	**Oxi**	**Red**
K_d_, μM	7.9 ± 1.8	10.7 ± 1.3	>>102 ± 264	4.54 ± 0.45

## Discussion

Considering the role of TRs in diseases such as resistance to thyroid hormone (RTH), thyroid cancers, dwarfism and general metabolic disorders (12), attributed to some of their non-classical actions, we aimed to screen other possible interactions that TRs might exhibit inside a cell. Taken together, our results lead us to conclude that: (i) PDIA1 is a new interaction partner for both TR isoforms and is able to bind these receptors at *in vitro* and cellular levels; (ii) T3 hormone plays a minor role in this interaction; (iii) PDI is able to regulate the transcription of selective genes through TRα and TRβ in different ways, depending on the isoform and promoter region involved; and (iv) although PDI did not directly act in coregulator recruitment, it improves binding of TRβ to coactivators, and this binding appears to be under the modulation of disulfide bonds formed specifically in β isoforms, but does not impair activity of TRα.

Among the many different techniques that are used to discover new protein interactions, yeast two-hybrid screening (y2h) was chosen due to its robustness, simplicity, agility and capacity to identify binary interactions (53). We screened only the TRβ isoform due to its facility to express in different cell types and standardization. The TRβ human gene was easily subcloned into the y2h assay vector pBTM and we found a large amount of new interactions for TRβ and two already known ones. These four interactions confirmed the efficiency of the assay, while the almost 50 new interactions opened an unexpected range of new TR partners and mechanism of actions to be confirmed and further studied. Most importantly, this TRβ screening led us to the interaction with PDIA1 (26, 27, 54).

Evidence from the NR and PDIA1 relationship came from two observations. One is that PDIA1 can bind hormones such as estradiol (E2) and T3 (16, 25) due to the presence of two binding sites with comparable affinity for T3 (4.3 ± 1.4 μM); one of these overlaps with the estradiol binding site (25). In addition, it was reported that both hormones, E2 and T3, when bound to PDIA1, did not inhibit its chaperone or catalytic activity, making this protein a possible “reservoir” of hormones without lost activity (25, 55). The second is that PDIA1 has been proven to be not only an endoplasmic reticulum-related protein (56) but also is found in different cellular compartments, such as the nucleus (32, 57, 58).

To confirm that PDIA1 could bind both TR isoforms with or without T3, we performed two different assays. First, we confirmed the TRs—PDIA1 interaction inside 293T cells through coimmunoprecipitation assays. Our results showed that PDIA1 coimmunoprecipitated with TRα and TRβ independent of the presence of T3. Therefore, this interaction was assured in two different cell systems: yeast and human embryonic cells (293T).

**Figure 3:**
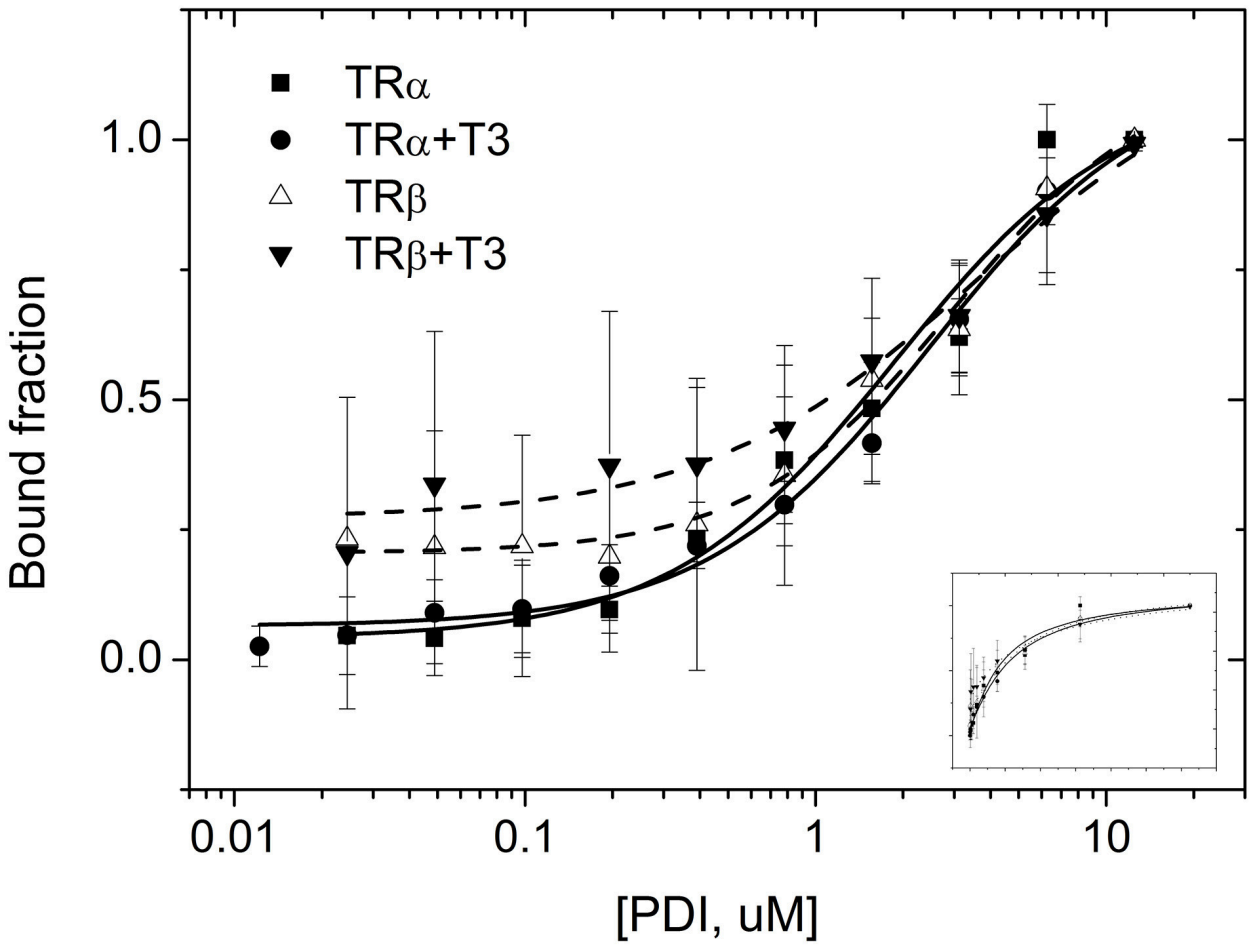
Fluorescence anisotropy curves of TRs binding to PDIA1 in the presence or absence of T3. TRα, as well as TRβ, binds to PDIA1 with similar affinity, and T3 makes no difference in binding affinities (Kd). Insert, same fluorescence plot, without logarithm scale in the x-axis.

**Figure 4:**
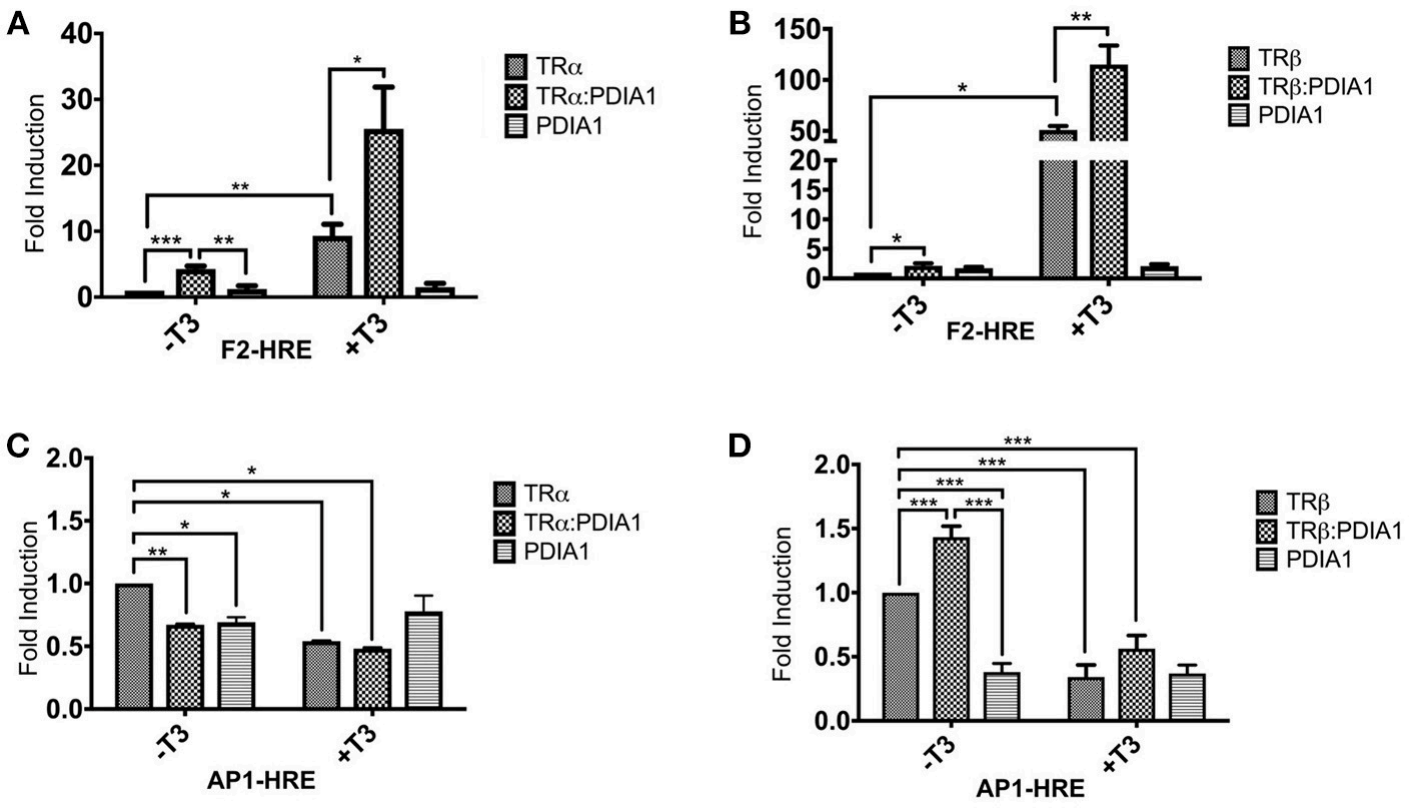
Reporter gene luciferase assay shows PDIA1 acting on TRs gene regulation. Full length TRα or TRβ, full length PDIA1 and Responsive Elements (REs) F2 and AP1, were transfected in 293T cells (in the presence and absence of T3), then luciferase activity was measured. All the TRs activation values were normalized by *Renilla* Luciferase activation. **(A)** In F2-Luc Response element, PDIA1 increased the basal activation of TRα in 4 times (^*^), and the T3 presence, which already increased TRα basal activation 9-fold (^**^), increased the TRα:PDIA1 activation 3-fold higher (^**^), or 25 times the TRα basal activity. **(B)** PDIA1 presence, in F2-Luc Response element, increased basal activity of TRβ in 2-fold (^*^) independently of T3 presence, the T3 increased TRβ activity in 50-fold (^***^), and PDIA1+T3 addition increased this activation in 2-fold (^***^), which in total, reached 115 times the basal activity of TRβ. **(C)** In AP1-Luc Response element, T3 repress basal activity of TRα, PDIA1 presence was able to decrease the basal gene expression in both cases, with (^*^) and without T3 (^**^), and T3 and PDIA1 together were able to halving the unliganded TRα activation (^*^). **(D)** In AP1-Luc Response element, when PDIA1 is present and T3 is not, basal activation increased 1.5-fold (^***^), and presence of T3 decreased even more this basal TRβ activation (about 60%) (^***^). However, both PDIA1 and T3 together maintained the basal activation also repressed but in lower level (about 40%) (^***^). In this last case, PDIA1 and T3 seemed to work in opposite directions. Statistical Analysis made with One-way or Two-way ANOVA ^*^p < 0.05, ^**^p < 0.01, ^***^p < 0.001.

Before performing the second interaction assay, we expressed and purified PDIA1, TRα and TRβ. Through the data obtained from DLS and SEC-GF (Supplementary Figure 3, Supplementary Table 2), we assumed that TRα and TRβ were eluted from SEC-GF as monomers with a R_h_ ~3.0 nm, as reported previously (36). Additionally, the complexes between each TR isoform and PDIA1 were formed and stabilized, according to data found in DLS and SEC-GF, which agreed with other TR complex formation data (39). Moreover, we performed a fluorescence anisotropy binding assay, and through this assay (Supplementary Figure 3), our results showed that PDIA1 could bind both TR isoforms, independently of the presence of T3 with reasonable affinity in a reduced environment. The measured K_d_ was ~2.0 μM for both TR isoforms, which represents a relatively strong interaction, as previously found for the interaction between TR and GATA2-Zf (38). So far, it seems that hormones and isoforms played no important role in the *in vitro* TR:PDIA1 relationship. Although one of our hypotheses was that PDIA1 might bind to T3 and directly deliver it to TR, helping in its action, we observed in co-IP (Figure 2), affinity assays (Figure 3), and reporter gene (Figure 4) experiments that the hormone is not essential for TR:PDIA1 interactions, and we discarded this hypothesis.

In addition, we investigated whether TR:PDIA1 interactions might influence TR gene regulation, as reported for estrogen hormone receptor α (ERα) (32), by reporter gene assays (Figure 4). Our results primarily showed that PDIA1, in general, upregulated the transcription of the luciferase gene in all conditions through F2 HRE (Figures 4A,B). This may highlight a mechanism of action based on two different functions: chaperone-like or as coactivator-like, similarly to some other proteins, such as Dot1L (59), TET3 (60), and PGC-1α (61).

Interestingly, we also observed very subtle differences in isoform modulation by PDIA1. One of these differences regards positive elements (F2, Figures 4A,B), in which PDIA1 increased basal activation of TRα, which was 2-fold stronger than basal activation of TRβ. Additional evidence for the differential modulation of isoforms was observed in negative elements (AP-1, Figures 4C,D), in which the presence of PDIA1 provoked antagonistic effects between TRα and TRβ, decreasing the former by near 30% and increasing the later by 50%. These results suggested that PDIA1 should play differential roles in isoform basal activation, probably interfering a corepressor dissociation and coactivator recruitment. More specifically, all data collectively suggest that corepressor dissociation might affect more TRα isoforms, and coactivator recruitment may be facilitated for TRβ in the presence of PDIA1.

On the other hand, it is important to mention that the role of nuclear receptors in negative response elements (nHRE) is still not entirely clarified. Taking the already studied GATA2-TR interaction as an example, it has been shown that T3 is able to weaken TR-nHRE interactions, which results in gene downregulation (36). However, in AP1 nHRE, T3 acted in both isoforms by downregulating luciferase expression, as expected. Interestingly PDIA1 presence *per se* was capable of downregulating the reporter gene only for TRα. Therefore, following this line, PDIA1 seems to have some comparable effects to those of GATA2-TR+T3 for this isoform and might be dissociating TRα from its nHRE. However, if PDIA1 is truly interfering in coregulator binding, this could be a better explanation of why PDIA1 derepressed TRβ gene regulation in the AP1 site. In fact, NR coregulators were recently described as playing different roles depending on each transcription factor or promoter region that they regulate by either enhancing or blocking the transcription (51). This mechanism is also known as the reversal role of coregulators, which could be an explanation for why PDIA1 acts by increasing or derepressing gene expression in this particular case.

**Figure 5:**
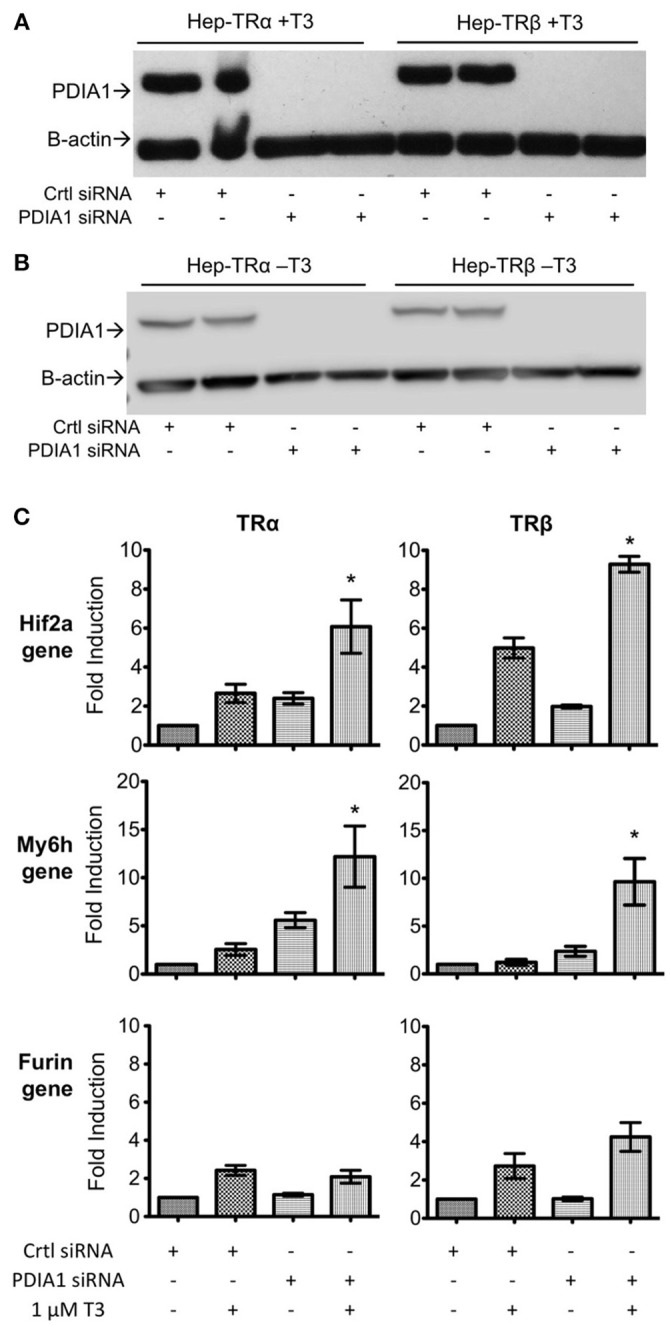
Knockdown of PDIA1 in Hep-TRα and Hep-TRβ cell lines followed by qPCR. **(A)** Knockdown of PDI in Hep-TRs cell lines in T3 presence (+T3). Western blot shows the absence of PDIA1 protein in knockdown samples (PDIA1 siRNA). Cells were treated with 1 μM of T3 for 6 h prior to cell harvesting. We presented here only 2 out of 3 knockdown experiments for each condition. Antibodies: anti-PDI and anti-β-Actin as loading control. **(B)** Knockdown of PDI in Hep-TRs cell lines in T3 absence (–T3). Western blot shows the absence of PDIA1 protein in knockdown samples (PDIA1 siRNA). Cells were treated with 1 μM of DMSO for 6 h prior to cell harvesting. We presented here only 2 out of 3 knockdown experiments for each condition. Antibodies: anti-PDI and anti-β-Actin as loading control. **(C)** qPCRs for each cell line, Hep-TRα and Hep-TRβ, with or without T3. Hif2a and Myh6 in absence of PDI (knockdown condition), increases transcription, Furin in absence of PDI (knockdown condition), did not alter gene expression. Each experiment was performed in three biological replicates. Statistical Analysis with One-way ANOVA ^*^p < 0.05.

Regarding gene regulation, we performed knockdown of PDIA1 in HepG2-TR cell lines (Figure 5). Two of the selected genes, Hif2a and Myh6, presented the same expression profile in which PDIA1 repressed their expression. This is additional evidence that PDIA1 might interfere in coregulator recruitment for both genes but represses transcription. Again, here we observed that repression effects seem to be more pronounced in TRα isoforms. In addition, PDIA1 absence had no relevance for the furin gene, which has no significant increase or decrease in its expression, revealing that its regulation might be promoter specific. So far, these results suggest two more perceptions on PDIA1 intervention in TR gene regulation. First, that PDIA1 action might be selective for each gene regulated by TR and, second, that the variety of regulating regions present in each gene promoter might be another factor that contributes to PDIA1 action.

**Figure 6:**
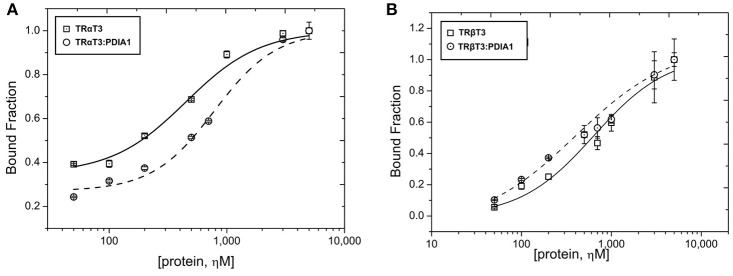
Fluorescence anisotropy of liganded TRs or complex (TR+T3:PDIA1) on SRC1 coactivator. **(A)** Anisotropy curves of TRα+T3 with and without PDIA1 titration in 50 nM of SRC1 peptide. **(B)** Anisotropy curves of TRβ+T3 with and without PDIA1 titrated in 50 nM of SRC1 peptide. These curves represent the average of the triplicate.

**Figure 7:**
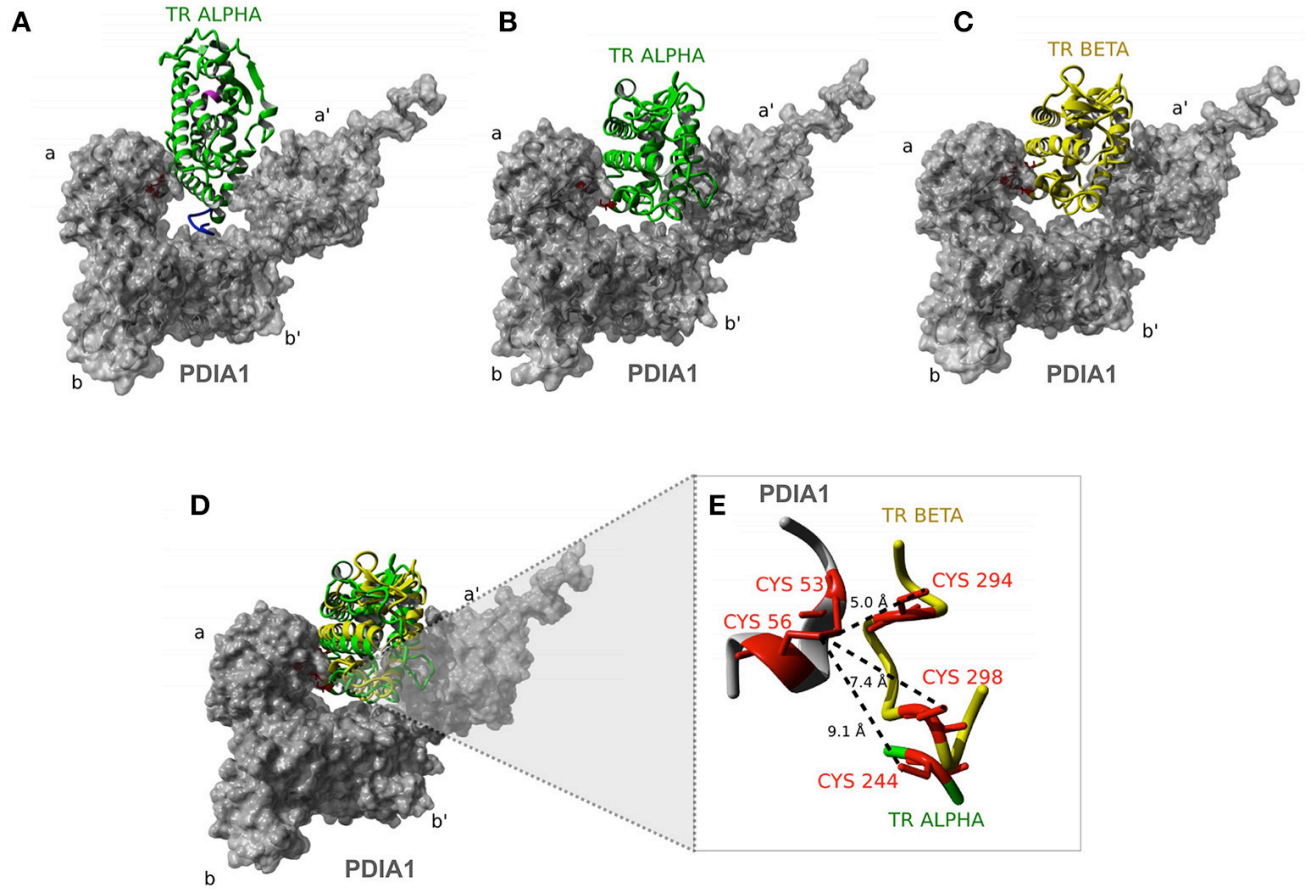
Docking analysis of TR:PDIA1 binding sites. **(A)** Model I of TRα: PDIA1 interaction. Structural representation showing the interaction between TRα (green) and PDIA1 (surface presented in gray). The a, b, a′, and b′ Domains are shown in PDIA1 structure. In pink we show H12 helix. Hinge Domain of TRα is shown in blue. This domain interacts with domain b′ of PDIA1. **(B)** Model II of TRα:PDIA1 interaction. Cysteines 53 and 56 of PDIA1 are close to cys244 in TRα, located in H5. In pink we show H12 helix. **(C)** TRβ:PDIA1 interaction (similar to TRα:PDIA1 Model II). Structural representation showing the interaction between TRβ (yellow) and PDIA1 (surface presented in gray). In pink, the H12 helix. **(D)** Overlap of TRα and TRβ models **(B,C)**. **(E)** Detail of PDIA1 cysteines and TRα and TRβ cysteines. Interaction between 53 and 56 cysteins of PDIA1 (red in gray) and 294 and 298 cysteins of TRβ (red in yellow) and 244 cysteine of TRα (red in green). Dotted lines show the distance between thiol groups of each protein.

To determine whether PDIA1 might act in TR-coregulator recruitment, by modification of their binding mode, or in their binding interfaces, we performed coactivator recruitment assays (Figure 7). Our results showed that PDIA1 presence did not affect overall coactivator binding, since the K_d_s are in the same order of magnitude in the presence or absence of PDIA1. However, in a deep analysis, we observed that PDIA1 decreased affinity for TRα:SRC1 complex formation to 50% and improved the binding of TRβ:SRC1 in the same proportion. This reinforced the hypothesis that PDIA1 has some influence on TR isoform activation, more specifically regarding roles in coactivator recruitment.

In sequence, we built a computational model to observe possible interaction interfaces that are most energetically favorable in the complex formed between TR and PDIA1. Based on docking analysis of TRα and β and PDIA1, we hypothesized about regions that were involved in the complex formation and about isoform differences in complex formation. As expected, a disulfide isomerase enzyme works through its active cysteines, but the proximity of those cysteines might result in reducing, oxidizing, and isomerizing disulfide bonds. In this way, we investigated whether PDIA1 might have some of these effects. Based on the three generated models, we observed the first dramatic difference between both isoforms: while TRα presented two possible binding modes to PDIA1, TRβ showed only one possibility for docking. Moreover, this unique binding mode for TRβ was the same as “TRα model 2,” indicating that this conformation is equally possible in both isoforms but is more stable for the β isoform. This higher stabilization provided for the TRβ:PDIA1 complex may be a consequence of the presence of two cysteine residues next to each other, which are present in TRβ (Cys 298 and Cys 294) but not in TRα. In addition, these two TRβ residues are close enough to PDIA1 cysteines (Cys 53 and Cys 56), as it was shown in our model in Figure 7, which may possibly provide the correct formation for TRβ disulfide bonds. In more detail, these cysteines are located in the H4-H5 position, and it is notable to assume that a disulfide bond in this region might lead to a better interaction with coregulators, such as SRC1. In this way, it seems that PDIA1 may present redox isomerase activity for TRβ, as it was reported for Ref-1 (62, 63) and GH (34).

**Figure 8:**
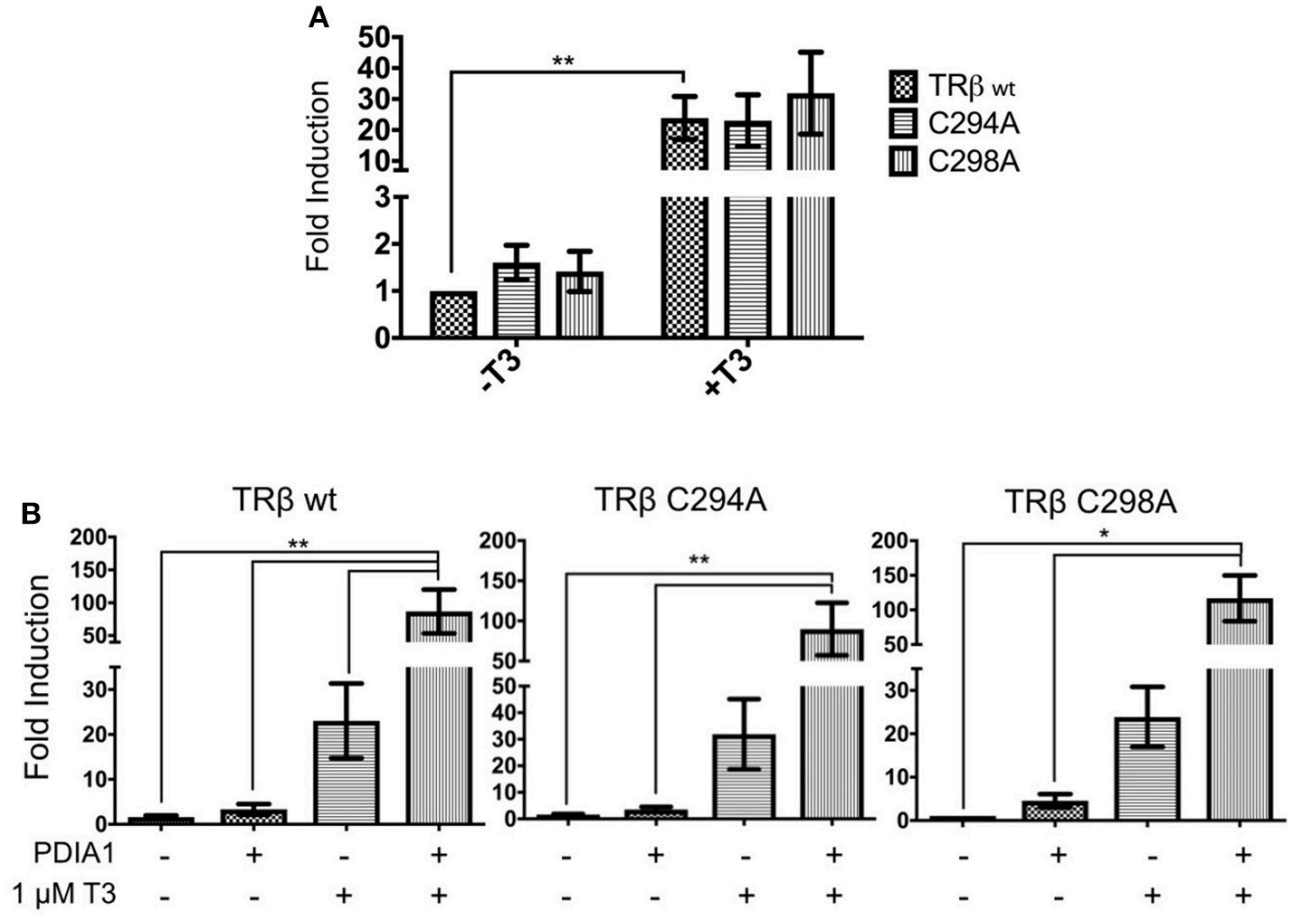
Reporter gene luciferase assay shows PDIA1 acting on TRβ gene regulation. Full length TRβ wt, C294A, and C298A, full length PDIA1 and Responsive Element (RE) F2, were transfected in 293T cells (in the presence and absence of T3), then luciferase activity was measured. All the TRs activation values were normalized by *Renilla* Luciferase activation. **(A)** First, T3 hormone addition in all cases increased the transactivation, this shows that in terms of gene expression, both TRβ mutants were activated in a similar way. **(B)** PDIA1 presence in the TRβ mutant transactivation assay led to increase TRβ-C294A and TRβ-C298A basal activation by about 3- to 4-fold. On the other hand, in the presence of T3, PDIA1 promoted minor further reduction in TRβ-C294A and TRβ-C298A activation in comparison with wt TRβ (86-fold for C294A, 89-fold for C298A, and 115-fold for TRβ wt). Statistical Analysis with One-way or Two-way ANOVA ^*^p < 0.05, ^**^p < 0.01.

To investigate the possible roles of TRβ cysteines in TR—PDIA1 interactions, we tested whether PDIA1 cysteines were important for TRβ transcriptional activity. So far, we made single-point mutations in TRβ (C298A and C294A), and the C294A mutant was more similar to TRα in terms of sequence and cysteine residues. Nonetheless, our reporter gene assay (Figure 8) indicated that the mutations did not significantly change TRβ activation by T3. The PDIA1 presence still increased TRβ activation, but to a lower degree. In other words, only one cysteine was enough to provide increased gene transcription under PDIA1 modulation, as it happened in TRα and in TRβ mutants (C298A and C294A). However, the presence of two cysteines appears to better stabilize the receptor, thereby increasing gene transcription, which may reflect the oxidoreductase/isomerase activity of PDIA1 in this complex. Finally, we also observed that the change in the redox state of PDIA1 modifies its affinities for TRβ binding but not for TRα binding. These results confirmed the preference for TRβ binding to PDIA1 and suggested that the PDIA1 redox state is important for interactions with this receptor isoform. Moreover, our results indicated that a reduced environment favors the PDIA1—TRβ interaction, suggesting that redox mechanisms might be involved in these protein interactions. Altogether, these results indicated that PDI regulates TRs through a different mechanism, which may involve disulfide bond formation in β isoforms of this NR and might be related to a redox state of TR and PDIA1.

**Figure 9:**
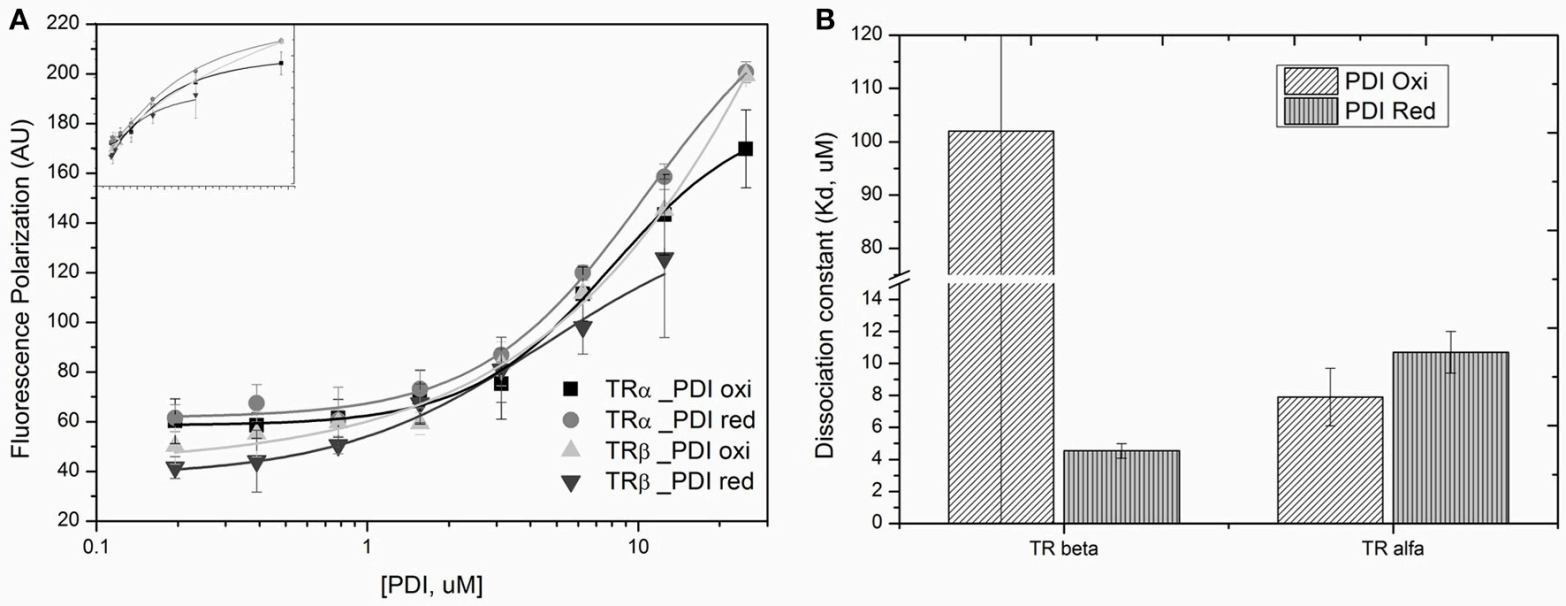
Binding profile for TRs binding to PDIA1 under different redox state. **(A)** Anisotropy curves of PDIA1 titrated under TRα and TRβ labeled with FITC. **(B)** Binding constants found for TRα and TRβ bound to reduced or oxidized PDIA1 (PDI red or PDI red).

In summary, here we present PDIA1 as a new interaction partner for both TR isoforms, which are able to bind these receptors at different levels. In addition, T3 did not modify this interaction; while PDI is able to regulate the transcription of TRα and TRβ selective genes, depending on the isoform and promoter region involved. Moreover, despite PDI did not directly act in coregulator recruitment, our results indicate that it improves TRβ binding to coactivators, and that this binding is under the modulation of disulfide bonds formed specifically in β isoforms, but does not impair activity of TRα.

## Author Contributions

AF: conception and design of the study. JC, NV, HR, FB, JF, NI, and MN: experiments and data collection. JC, AF, NV, TD, NI, HR, AC, MB, and PW: data analysis. JC, AF, NV, NI, HR, PW, and FL: drafting and critical revision of the paper. JC, AF, PW, and FL: final revision to be published.

### Conflict of Interest Statement

The authors declare that the research was conducted in the absence of any commercial or financial relationships that could be construed as a potential conflict of interest.

## Abbreviations

TR: Thyroid Hormone Receptor
PDIA1 or P4HB: Protein Disulfide Isomerase A1 or Prolyl 4-Hydroxylase Subunit Beta
NR: Nuclear Receptor
Co-IP: Co-Immunoprecipitation
T3: Triiodothyronine
T4: Thyroxine
TH: Thyroid Hormones
NT: N-terminal Transactivation Domain
DBD: DNA Binding Domain
LBD: Ligand Binding Domain
TRE: Thyroid Hormone Responsive Elements
CTBP: Cytosolic T3-Binding Protein
NADPH: Nicotinamide adenine dinucleotide phosphate
ER: Estrogen Hormone Receptor
GH: Growth Hormone
Ref-1: Redox Factor-1
GFP: Green Fluorescent Protein
y2h: yeast two-hybrid
3-AT: 3-amino-1,2,4-triazole
WB: Western Blot
SEC: Size Exclusion Chromatography
CV: Column Volume
SEC-GF: Analytical Gel Filtration
DLS: Dynamic Light Scattering
PdI: Polydispersity Index
FITC: fluorescein isothiocyanate
Kd: Affinity Constant
pRL: *Renilla reniformis* luciferase
qRT-PCR: quantitative Real Time PCR
SRC1: nuclear receptor coactivator 1
PDB: Protein Data Bank
PPI: Protein-Protein Interaction.
