# Supplementary material for: Protein Disulfide Isomerase Modulates the Activation of Thyroid Hormone Receptors
Source: Front Endocrinol (Lausanne). 2019 Jan 8;9:784. doi: 10.3389/fendo.2018.00784 (PMC6331412; doi:10.3389/fendo.2018.00784)

Supplemental material - RESEARCH ARTICLES

Protein Disulfide Isomerase modulates activation of Thyroid Hormone Receptors

Jessica L. O. Campos[1,2]; Tabata R. Doratioto[1,2]; Natalia B. Videira[1,2]; Helder V. Ribeiro Filho[1,2]; Fernanda A. H. Batista[1]; Juliana Fattori[1]; Nathalia de C. Indolfo[1,2]; Marcel Nakahira[3], Marcio Chaim Bajgelman[1], Aleksandra Cvoro[4]; Francisco R. M. Laurindo[5]; Paul Webb[6]; Ana Carolina M. Figueira[1].

Supplementary Table 1. Primers used in qPCR assays.

**Primer**	**Direction**	**Sequence**
PDIA1	Forward	CATCGTGAACTGGCTGAAGA
PDIA1	Reverse	CTCCACGTCCTTGAAGAAGC
FURIN	Forward	ACAACTATGGGACGCTGACC
FURIN	Reverse	TGGACACAGCTCTTCTGGTG
HIF2A	Forward	CCACCAGCTTCACTCTCTCC
HIF2A	Reverse	TCAGAAAAAGGCCACTGCTT
MYH6	Forward	CCACCCAAGTTCGACAAGAT
MYH6	Reverse	CACAGAAGAGGCCCGAGTAG
GAPDH	Forward	ACCTGCCGCCTGGAGAAACC
GAPDH	Forward	GACCATGAGGTCCACCACCCTG

Supplementary Table 2. Size parameters – hydrodynamic radius (Rh), and molecular weight – of TRα, TRβ and PDIA1 and of TRα:PDIA1 and TRβ:PDIA1 complexes determined by Analytical Gel Filtration (GF) and Dynamic light scattering (DLS).

**Protein**	**R_h DLS (nm)**	**R_h GF (nm)**	**GF elution (mL)**	**MW GF ***	**MW theoretical ***
TRα	3.2	2.8	15.86	40.000	47.000
TRβ	3.2	2.9	16.52	40.000	42.000
PDIA1	3.3	3.2	14.94	52.000	57.000
TRα:PDIA1	4.2	4.0	13.78	104.000	104.000
TRβ:PDIA1	4.2	3.8	13.83	87.000	99.000

*unit of mass corresponds to 1/12 of one atom of p1 carbon 12

Supplementary Figure 1. Activation confirmation of the TR constructs in LV-flagTR-Ig vector used in cell experiments. A. Western Blot shows transfection and expression of flag-tagged TRs in 293T cells. The expression of both TR isoforms decreased after 36 h of T3 treatment, and β-actin control show the amount of protein loaded into each well. Negative control of western blot is 293T cell extract without transfection. The quantification of bands are presented in the chart beside the western blot. B. Luciferase Gene Reporter show the transactivation of flag-TRs in the presence of T3. In F2 response elements, it is possible to observe 4-7 fold increase, and in DR4 element, 1.5-3 fold, after T3 treatment. These assays comprove that flag tagged TR constructions are still active, even after 36h of T3 treatment.

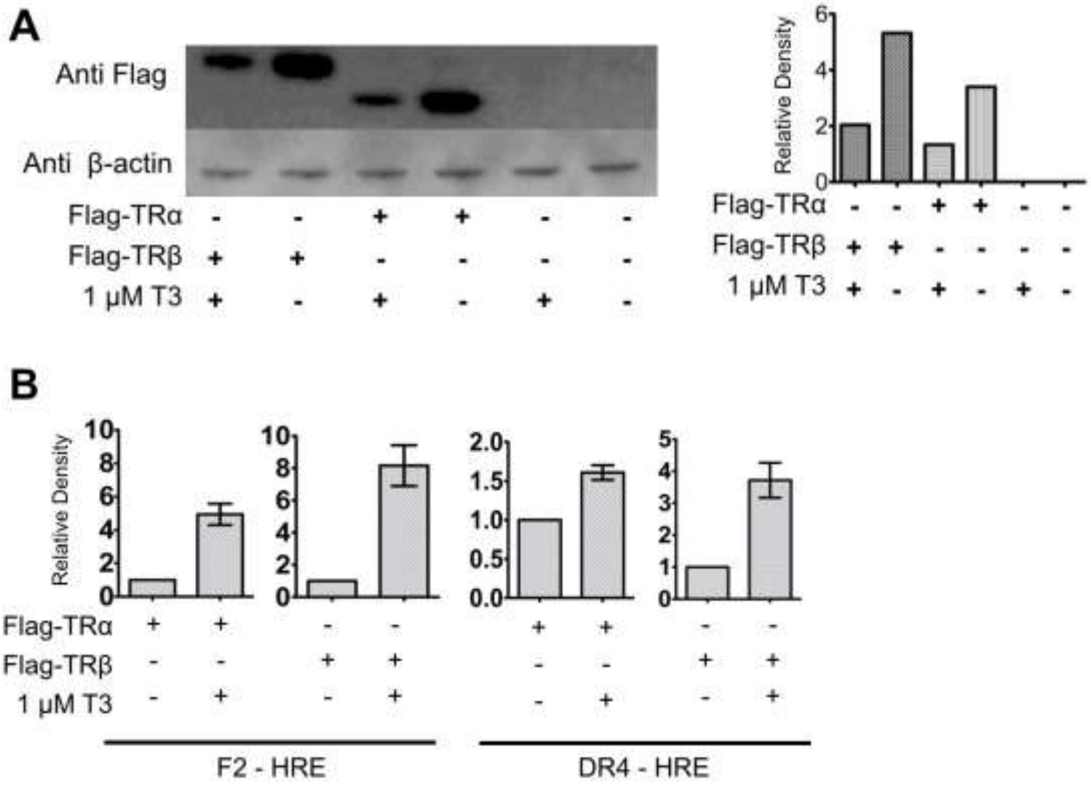

Supplementary Figure 2. Co-immunoprecipitation of TR:PDIA1 in 293T cells shows the interaction for both isoforms with or without T3 presence. A. Control experiment for Co-IP TR:PDIA1 (Figure 2A) showing the input of exogenous expressed flag-TRα and flag-TRβ, and IP of 4 samples (TRα-T3; TRα+T3; TRβ-T3; TRβ+T3). B. Confirmation experiment of TR:PDIA1 Co-IP (Figure 2B, duplicate). Immunoprecipitation of flag-TRs followed by WB anti-flag and WB anti-PDIA1, showing the success of Co-IP. We found both bands of TR isoforms in IP lanes. Although the PDI bands are weak, we were able to see a small amount of PDIA1 in IP lanes, proving again that both proteins were co-immunoprecipitated.

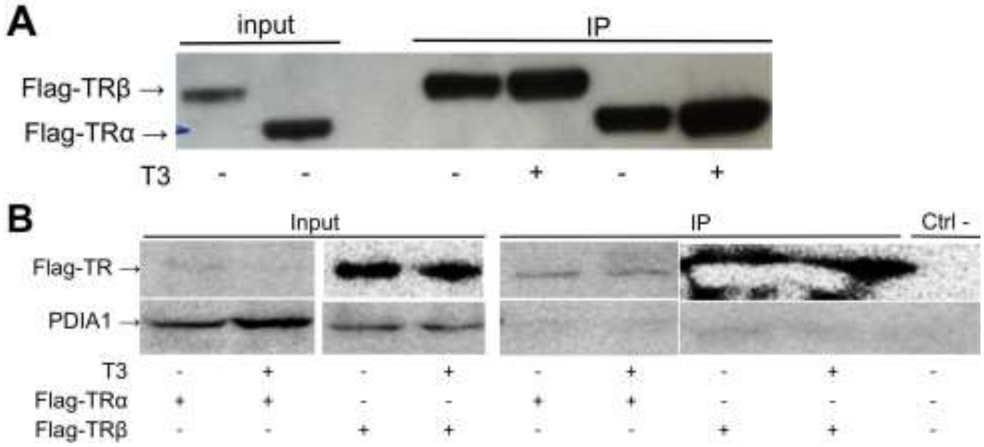

Supplementary Figure 3. Analytical Gel Filtration Chromatography (GF) profiles for isolated TRs and PDIA1 and in complex (Superdex 200 HR 10/300 size exclusion column 1 × 30 cm). Before the GF analysis the column was calibrated and standardized with the gel filtration calibration kits, which allowed us to calculate estimated hydrodynamic radii of our proteins. A. TRα (Rh - 2.8 nm), PDIA1 (Rh - 3.2 nm), and TRα:PDI complex (Rh - 4 nm). B. TRβ (Rh - 2.9 nm), PDIA1 (Rh - 3.2 nm), and TRβ:PDI complex (Rh - 3.8 nm). It is possible to observe that TRs eluted in the higher volumes, which corresponds to the lowest species, PDIA1 eluted with intermediary size and the complexes show the highest molecular weight.

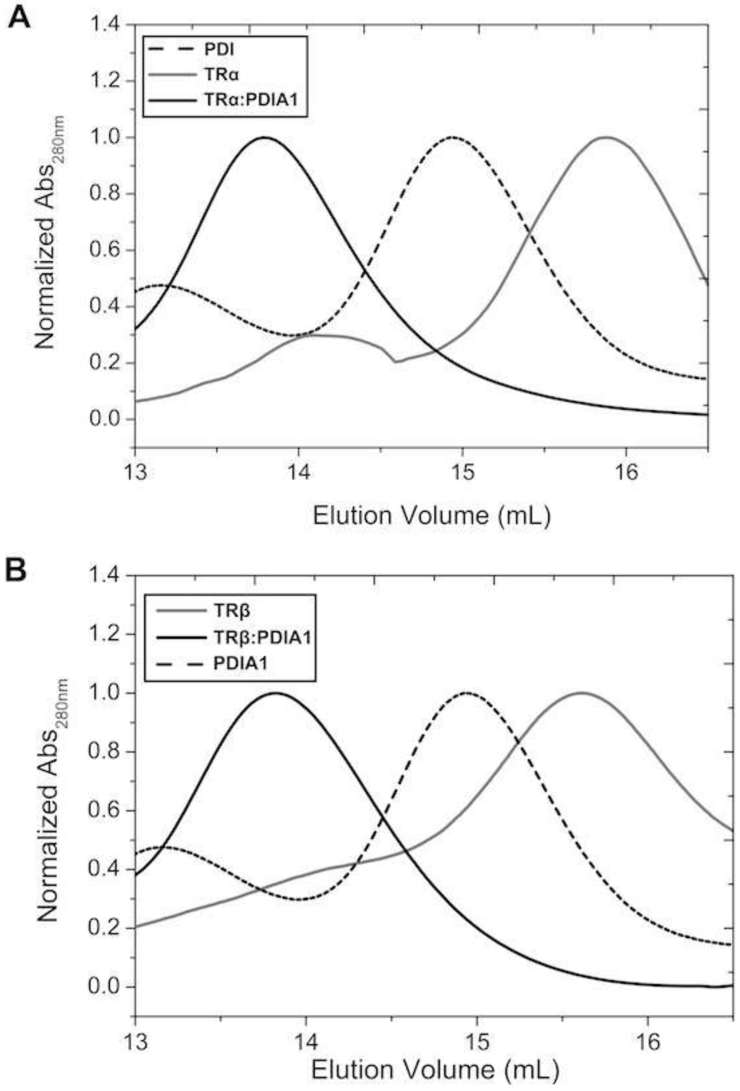

Supplementary Figure 4. Graphic quantification of western blot bands showing the knockdown in protein level of PDIA1 (Figure 6 in Manuscript). Antibodies: anti-PDIA1 and anti-β-Actin as loading control.

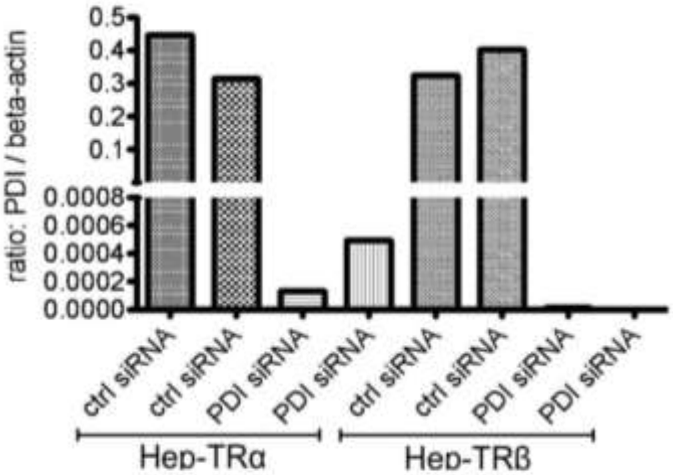

Supplement: Supplementary file 1 [file Data_Sheet_1.PDF]
